# Primary retroperitoneal nodal endometrioid carcinoma associated with Lynch syndrome: A case report

**DOI:** 10.3389/fonc.2023.1092044

**Published:** 2023-02-21

**Authors:** Daniela Fischerova, Umberto Scovazzi, Natacha Sousa, Tatevik Hovhannisyan, Andrea Burgetova, Pavel Dundr, Kristýna Němejcová, Rosalie Bennett, Michal Vočka, Filip Frühauf, Roman Kocian, Tereza Indrielle-Kelly, David Cibula

**Affiliations:** ^1^ Department of Obstetrics and Gynecology, First Faculty of Medicine, Charles University and General University Hospital in Prague, Prague, Czechia; ^2^ Department of Gynecology and Obstetrics, Ospedale Policlinico San Martino and University of Genoa, Genova, Italy; ^3^ Department of Gynecology and Obstetrics, Hospital de Braga, Braga, Portugal; ^4^ Department of Gynecology and Gynecologic Oncology, Nairi Medical Center (MC), Yerevan, Armenia; ^5^ Department of Radiology, First Faculty of Medicine, Charles University and General University Hospital in Prague, Prague, Czechia; ^6^ Department of Pathology, First Faculty of Medicine, Charles University and General University Hospital in Prague, Prague, Czechia; ^7^ Department of Oncology, First Faculty of Medicine, Charles University, Prague, Czechia; ^8^ Department of Obstetrics and Gynecology, Burton Hospitals National Health System (NHS), West Midlands, United Kingdom

**Keywords:** Lynch syndrome, hereditary nonpolyposis, ultrasonography, biopsy, adenocarcinoma, lymph nodes, genetic testing, immunohistochemistry

## Abstract

We report a rare case of primary nodal, poorly differentiated endometrioid carcinoma associated with Lynch syndrome. A 29-year-old female patient was referred by her general gynecologist for further imaging with suspected right-sided ovarian endometrioid cyst. Ultrasound examination by an expert gynecological sonographer at tertiary center revealed unremarkable findings in the abdomen and pelvis apart from three iliac lymph nodes showing signs of malignant infiltration in the right obturator fossa and two lesions in the 4b segment of the liver. During the same appointment ultrasound guided tru-cut biopsy was performed to differentiate hematological malignancy from carcinomatous lymph node infiltration. Based on the histological findings of endometrioid carcinoma from lymph node biopsy, primary debulking surgery including hysterectomy and salpingo-oophorectomy was performed. Endometrioid carcinoma was confirmed only in the three lymph nodes suspected on the expert scan and primary nodal origin of endometroid carcinoma developed from ectopic Müllerian tissue was considered. As a part of the pathological examination immunohistochemistry analysis for mismatch repair protein (MMR) expression was done. The findings of deficient mismatch repair proteins (dMMR) led to additional genetic testing, which revealed deletion of the entire EPCAM gene up to exon 1-8 of the MSH2 gene. This was unexpected considering her insignificant family history of cancer. We discuss the diagnostic work-up for patients presenting with metastatic lymph node infiltration by cancer of unknown primary and possible reasons for malignant lymph node transformation associated with Lynch syndrome.

## 1 Introduction

Patients with malignant infiltration of lymph nodes and negative history of malignancy represent a diagnostic challenge. The infiltration of lymph nodes can be related to metastases of cancer of unknown primary site (CUP) (1), lymphoproliferation (2), melanomas (3, 4), and others. There are also rare cases of carcinomas arising primarily from lymph nodes, associated with malignant transformation of ectopic epithelial tissue, such as carcinoma arising in endosalpingeosis (5).

CUP accounts for approximately 3–5% of all malignant neoplasms (6). It represents a heterogeneous group of metastatic tumors for which no primary site is detected following a full diagnostic work-up and in 30% of all CUP patients even at autopsy (6). More than 50% of CUP patients present with multiple sites of involvement, and the single site CUP are most commonly in the liver, lymph nodes, peritoneum, lungs, bones, and brain (6). Although iliac lymph node metastases are usually related to gynecological cancers (uterine cervix, endometrium, the tubes, and ovaries) or colorectal cancer, their involvement with unknown primary cancer in women is rare (7).

There are recommendations available regarding the diagnosis, treatment, and follow-up of CUP (8, 9). Primary tumor can be anticipated based on the regional drainage of the infiltrated lymph node(s). Obtaining histology from the infiltrated lymph nodes using tru-cut biopsy or fine-needle aspiration (FNA) can be done under ultrasound guidance. These initial steps help direct search for primary tumor and choose appropriate treatment strategy (7, 10–14). The recommended whole-body imaging for targeted search for primary source of tumor dissemination is positron emission tomography combined with computed tomography (PET-CT) or whole-body diffusion-weighted magnetic resonance imaging (15, 16). The addition of a more extended tumor marker profile can also be considered (16).

Less likely, *de novo* primary nodal malignant transformation has been reported in literature associated with malignant transformation of ectopic epithelial tissue, such as serous carcinoma arising in endosalpingeosis, for example, a case of a serous borderline tumor or a high-grade serous carcinoma within an inguinal lymph node without known primary tumor (17, 18).

The occurrence of carcinoma in younger patients can be associated with hereditary syndromes characterized by germline mutation with higher risk of cancer development. A possible source of endometrioid carcinoma, which can be associated with Lynch or Cowden syndrome, can be the uterus or ovary, or endometriosis in any localization (19). Hereditary breast and ovarian cancer (HBOC) syndrome is associated with no or low risk of endometrial cancer, however, of different histotype, mainly endometrial serous carcinoma (20, 21). Lynch syndrome is an autosomal dominant disorder caused by a germline mutation in one of several DNA mismatch repair (MMR) genes (mostly *MSH2*, *MLH1*, *MSH6*, and *PMS2*), and it is not only the most common cause of inherited colorectal cancer but it also accounts for approximately 3% of endometrial cancer (mainly endometrioid carcinoma often located in the lower uterine segment) (22–25). Atypical manifestations related to Lynch syndrome were also described in literature, as Lynch syndrome–associated squamous cell CUP in retroperitoneal lymph node (26) and primary peritoneal endometrioid carcinoma after prophylactic gynecological surgery (27). There was also a case of an incidental endometrioid carcinoma of unknown primary isolated in the external iliac lymph nodes at risk reducing hysterectomy and bilateral salpingo-oophorectomy in a Lynch syndrome carrier with no history other than previous hyperplastic polyp (28).

We describe a rare case of a 29-year-old female patient presenting with infiltrated lymph nodes in the right obturator fossa with no visible primary source.

## 2 Case summary

A 29-year-old patient was referred for an expert ultrasound examination to gynecologic oncology center for hypoechogenic round lesion, presumed endometrioma. The patient did not have any symptoms suggestive of endometriosis. Apart from two Caesarean sections and tonsillectomy, her surgical history was unremarkable. She suffered from multiple sclerosis treated with immunosuppressive therapy (Interferon β) and bicuspid aortal valve. Her grandmother died the aged of 50 of pancreatic cancer, and her great grandfather succumbed to colorectal cancer at unknown age.

**Figure 1:**
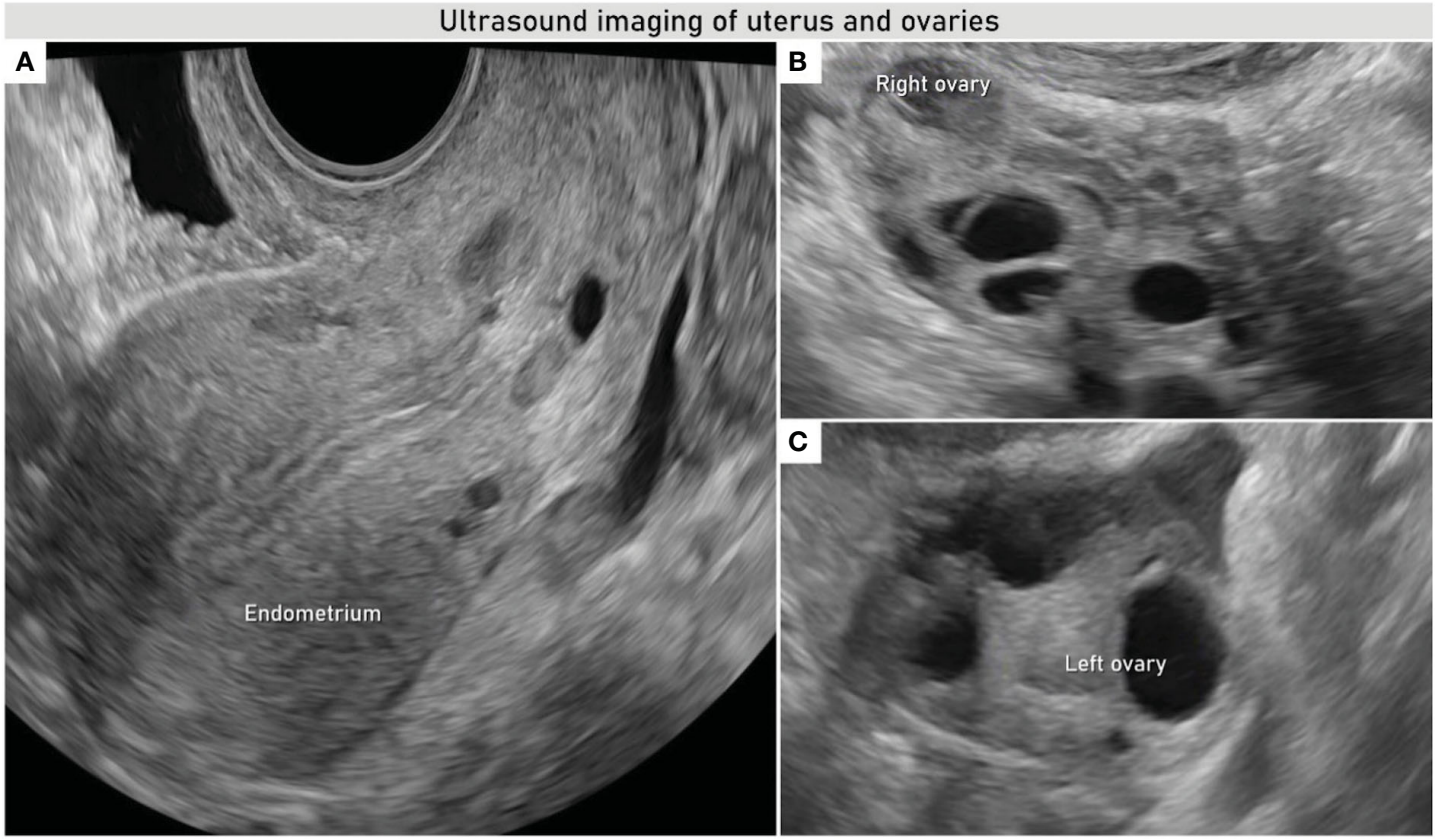
Transvaginal ultrasound evaluation of uterus and adnexa. Uniform endometrium **(A)** and right and left ovaries of normal appearance **(B, C)**.

**Figure 2:**
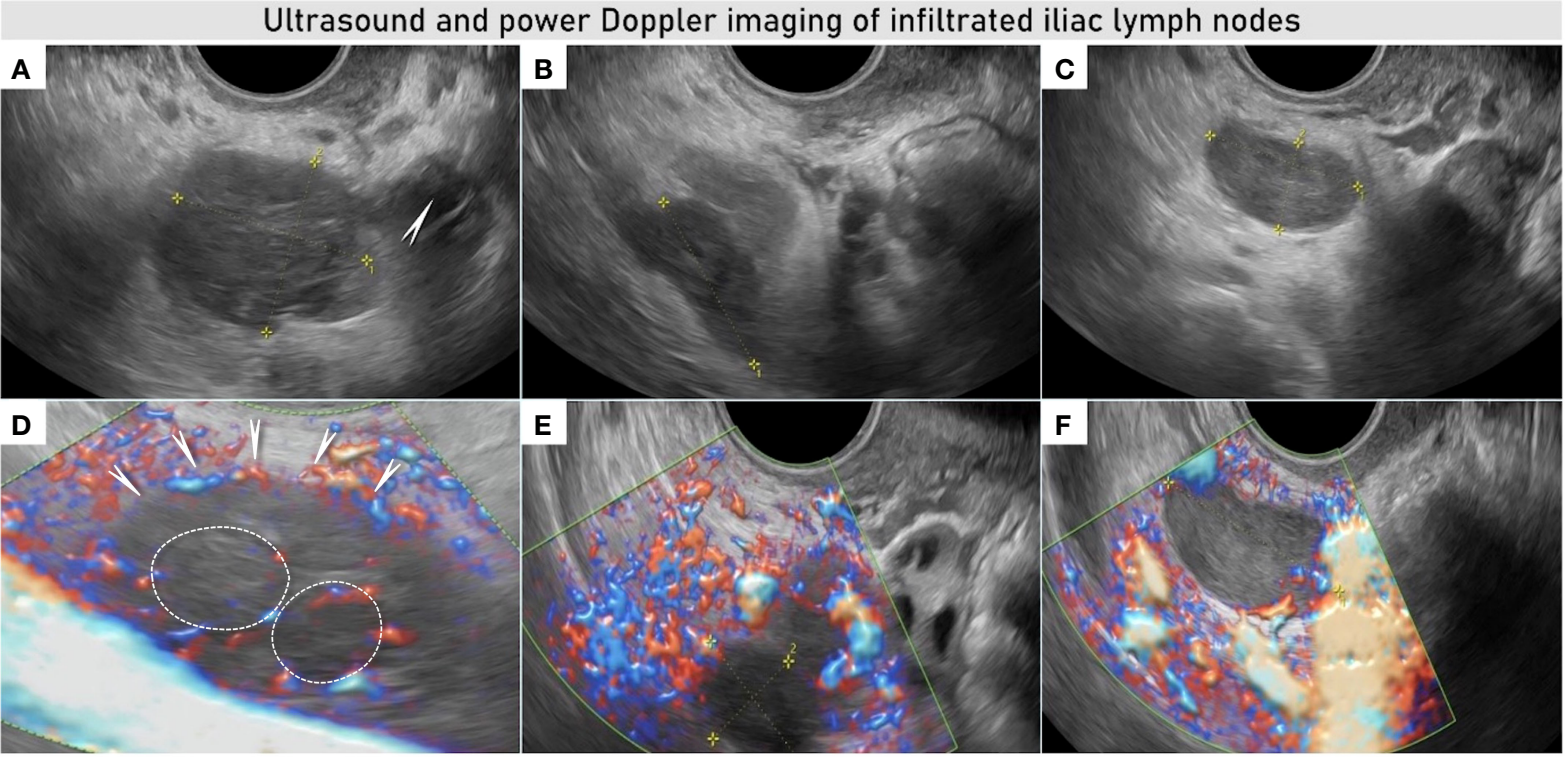
Ultrasound and power Doppler imaging of infiltrated lymph nodes in the right obturator fossa. Transvaginal ultrasound imaging demonstrates three bulky lymph nodes: first iliac lymph node (29 × 26 × 31 mm) infiltrated by tumor nodules located in the close proximity to the right ovary (arrow) **(A)** with visible transcapsular vessels penetrating the lymph node from the outside (arrows) and ring-shaped vessels marked with white circles **(D)**. Second iliac lymph node (25 × 13 × 21 mm) was lateral to the first one, infiltrating the pelvic side wall and internal iliac vessels up to the interiliac bifurcation with infiltration of the external iliac vein **(B)**, this lymph node was less vascularized on color Doppler images **(E)**. Third infiltrated node (26 × 13 × 30 mm) was located deeper in the pelvis, near the right lateral parametrium **(C)**, with transcapsular vessels on color Doppler image **(F)** See also **Videoclip 1**.

Ultrasound examination performed at gynecologic oncology center by experienced sonographer revealed normal gynecologic findings of the uterus and adnexa, smooth peritoneum, small amount of free fluid, and neither adhesions in the pelvis nor other signs related to endometriosis (**Figure 1**). Lateral to the right ovary, there were three bulky lymph nodes detected in the right obturator fossa, which were misinterpreted by the referring physician as endometrioma (**Figure 2** and **Videoclip 1**). Systematic ultrasound examination of pelvic and abdominal lymph nodes revealed no other suspicious lymph nodes in the retroperitoneum or groins. Expert sonographer detected two inhomogeneous lesions of 24 and 25 mm in the segment 4b of liver parenchyma, considered to be metastatic spread on gray scale ultrasound (**Supplementary Figure S1** and **Videoclip 2**). During the same visit, ultrasound-guided tru-cut biopsy of infiltrated lymph nodes was performed. To briefly describe ultrasound-guided tru-cut biopsy using a transvaginal approach, no patient preparation, fasting, or routine administration of analgesics or antibiotics is required for this minimally invasive approach in the outpatient setting (**Supplementary Figure S2** and **Videoclip 3**). The entire procedure is performed with the patient in the lithotomy position. The sonographer selects a safe site for biopsy from a lesion that is not necrotic or cystic, so that an adequate sample can be obtained for histologic analysis and immunohistochemical evaluation. After ruling out possible contraindications (in particular the use of anticoagulant drugs) and to ensure the safety of the procedure, a needle guide attached to the probe is used; a disposable needle (30 cm/18 Gauge) is inserted into the automated biopsy device, then the needle is inserted into the needle guide and the needle tip is inserted through the vaginal wall into the pelvis in close proximity to the infiltrating lymph nodes and aligned with the lesion. A penetration depth between 15 and 22 mm is selected, and the procedure is performed with continuous monitoring of patient comfort and needle position on the ultrasound machine monitor. Collection of two to three core samples allows for better adequacy and accuracy of histological analysis. The procedure takes several minutes. After biopsy, the biopsy site is checked for internal bleeding (“fountain sign”), and the intensity of the external per vaginal bleeding is checked. The patient is instructed and discharged home. The result of the histopathological examination is available within 72h (7, 10, 12).

In addition to the collection of the biopsy, a tumor marker profile was obtained during the same appointment and additional imaging tests were scheduled. Tumor marker profile included cancer antigen 125 (CA 125), carcinoembryonic antigen (CEA), cancer antigen 19-9 (CA 19-9), cancer antigen 15-3 (CA 15-3), and cytokeratine fraction 21-1 (CYFRA 21-1).The PET-CT was chosen for targeted search of primary tumor. Magnetic resonance imaging of the liver was added for closer characterization of liver lesions and their potential resectability.

**Figure 3:**
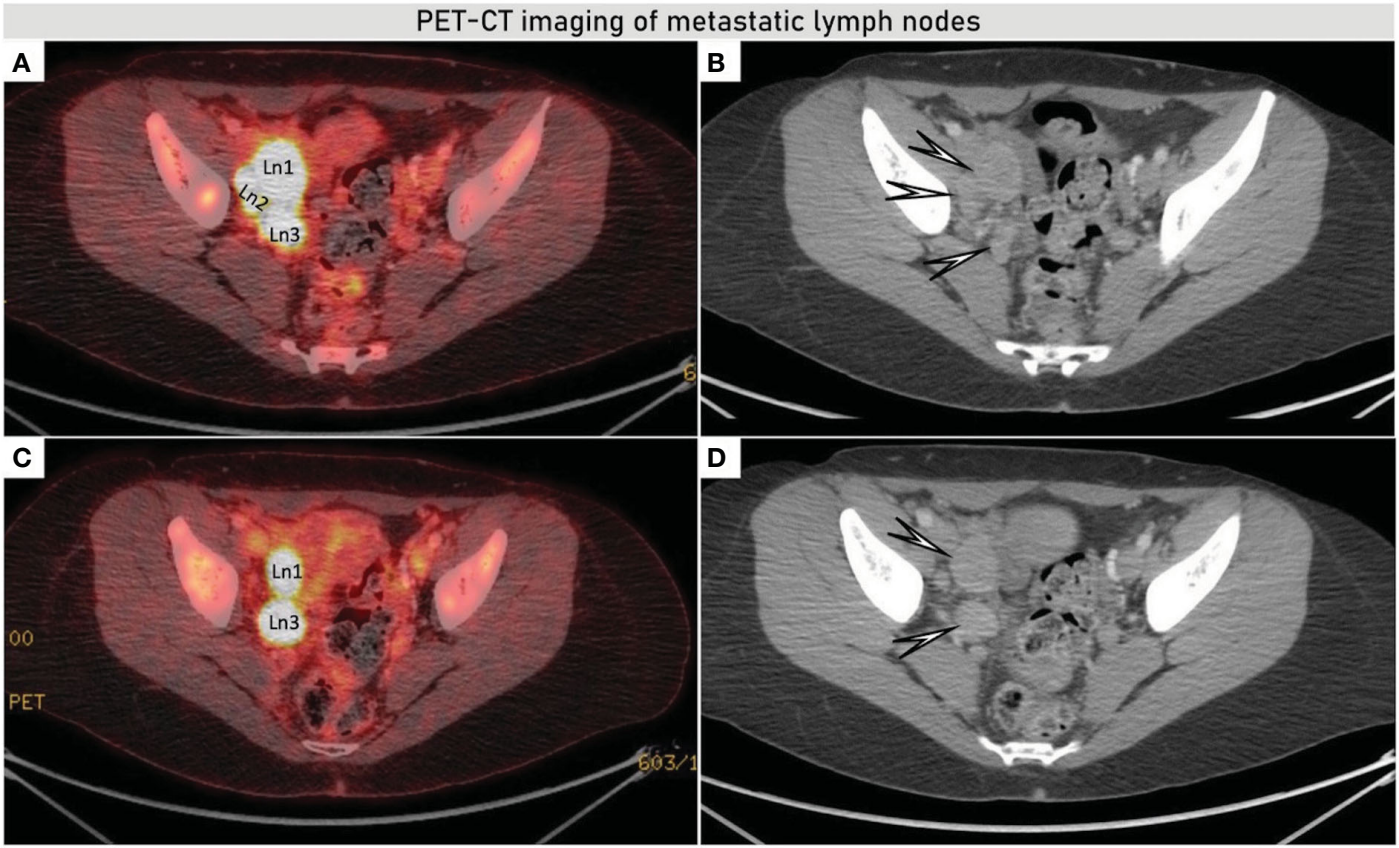
Axial fused PET-CT showed increased accumulation of 18F-FDG in the bulky iliac lymph nodes on the right obturator fossa of up to 36 × 25 mm **(A, C)**, morphologic images from CECT demonstrating enlarged nodes in the right obturator fossa (white arrows) **(B, D)**. PET-CT (positron emission tomography combined with computed tomography), CECT (contrast enhanced computed tomography), and 18F-FDG (18F- fluorodeoxyglucose).

Results were presented to the multidisciplinary team. Tumor markers CA 125, CEA, CA 19-9, CA15-3, and CYFRA 21-1 were in normal range. The PET-CT showed metabolic accumulation of 18F-fluorodeoxyglucose (18F-FDG) in the right obturator fossa (**Figure 3**) but no other metabolically active lesions in the body including the liver (**Supplementary Figure S1**). On MRI, the liver lesions showed enhancement in the early stage with persistence to late stage suggesting the lesions to be more likely adenoma or focal nodular hyperplasia (**Supplementary Figure S1**). Tru-cut biopsy revealed a poorly differentiated tumor composed of solid sheets of polygonal eosinophilic cells with marked nuclear pleomorphism, conspicuous mitotic activity, and necrotic areas (**Supplementary Figure S3**).

Immunohistochemically, the tumor cells showed positive expression of PAX8 and CK7, loss of expression of PTEN, ARID1A, and p53 wild-type expression. This immunoprofile suggested Müllerian differentiation favoring endometrioid or clear cell carcinoma, since loss of PTEN and ARID1A is found in 40 and 50% of these cases (29). The markers used for distinction between endometrioid and clear cell carcinoma showed ambiguous results. HNF1β was mostly weakly to moderately positive (strong positivity is more typical for clear cell carcinomas). In **Supplementary Figure S4**, positive expression of CK7 and HNF1β were demonstrated. Napsin A, which is used as another marker of clear cell carcinoma with intermediate sensitivity, was negative. On the other hand, ER and PR were negative, which favor a diagnosis of clear cell carcinoma (30). In addition, WT1 showed negativity as a marker used to rule out the derivation from adnexal serous carcinoma. The morphology and immunophenotype of the tumor were not quite characteristic for either clear cell or endometroid carcinoma. However, due to the immunohistochemical findings, the first differential diagnosis was poorly differentiated endometrioid carcinoma and second possibility was eosinophilic variant of clear cell carcinoma. Due to the absence of primary origin of invasive carcinoma on the imaging (including PET-CT), we considered either a primarily nodal tumour arising from endometriosis or occult spread from another source (e.g. from the endometrium or ovary).

Since the patient’s reproductive plan was complete and the possibility of fertility preservation was not considered, the treatment plan would not have differed if additional biopsy specimens, such as endometrial biopsy, had been performed before definitive surgery. Surgical treatment including hysterectomy and bilateral salpingo-oophorectomy with extirpation of the infiltrating lymph nodes and systemic dissection of the pelvic and paraaortic lymph nodes was chosen.

Intraoperative assessment revealed no suspicious findings except the enlarged lymph nodes in the right obturator fossa. These were infiltrating the right pelvic side wall, including right internal iliac vessels and partially external iliac right vein, psoas muscle as well as S2–S3 rami of the lumbosacral plexus. The extent of the infiltration was not evident in any preoperative imaging assessment. Therefore, the senior surgeon was requested to join the intervention, and a laterally extended endopelvic resection was performed on the right side, including resection of the internal iliac vein, partial resection of the external iliac vein, psoas muscle, periostium of the pubic bone, and S2–S3 rami (divisions) of the lumbosacral plexus. Total abdominal hysterectomy with bilateral salpingoophorectomy, pelvic, and paraaortic lymph node dissection were carried out with no visible residual tumor left at the end of surgery.

**Figure 4:**
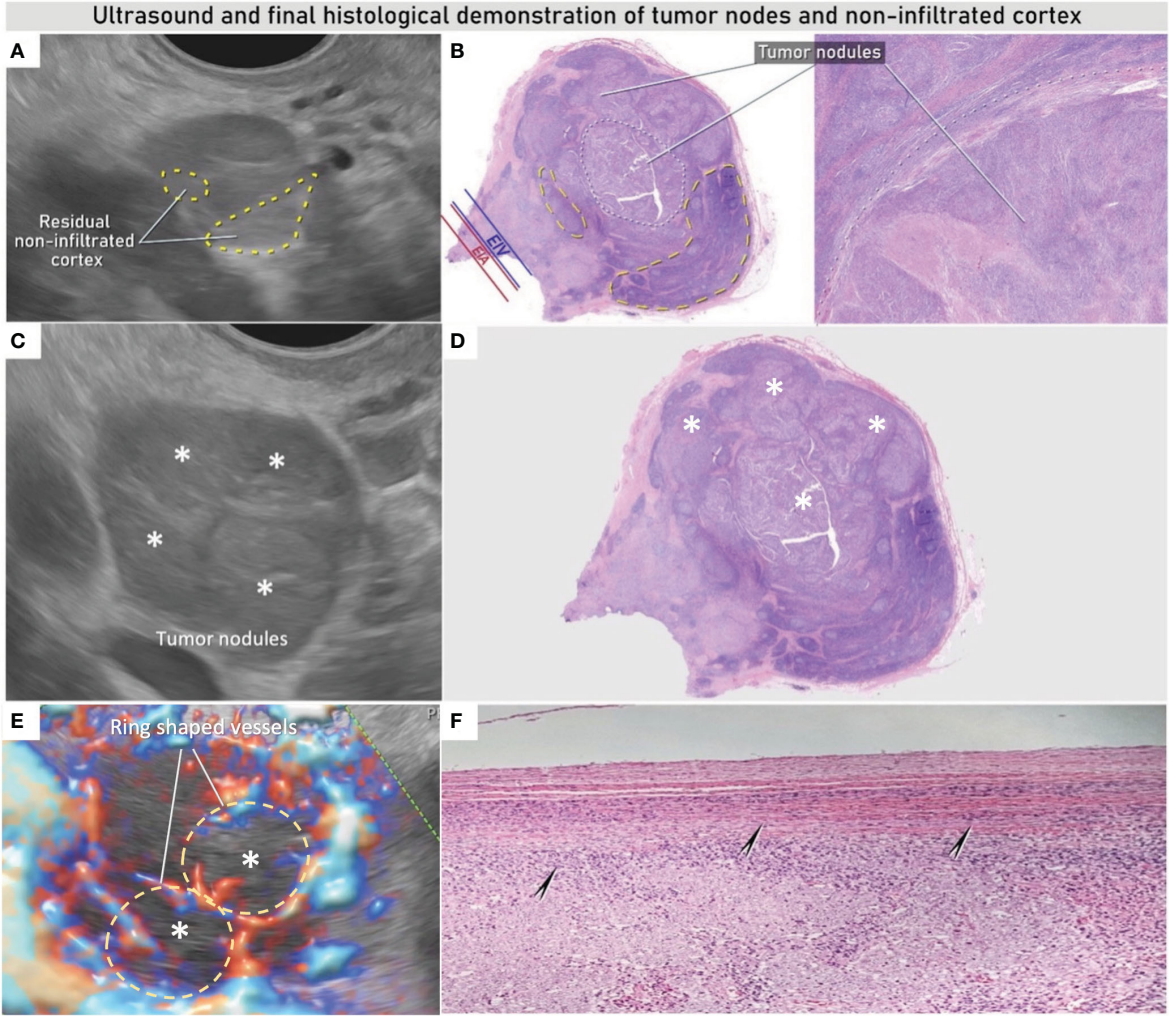
Ultrasound and final histological demonstration of infiltrated lymph node. Non-infiltrated residual cortex on the ultrasound and histological imaging of lymph node marked with yellow circles **(A, B)**, with intranodal involvement due to solid sheets of poorly differentiated extensively necrotic carcinoma marked with white circles on specimen **(B)** and white asterisks on ultrasound images **(C, E)** and on the specimen **(D)**; on Power Doppler imaging infiltrated nodules reveal a ring-shaped vascularization (yellow circles) in **(E)**; arrows indicate intact lymph node capsule **(F)**. See also **Videoclip 4**.

Histological evaluation of the final specimen from the uterus and adnexa did not reveal any cancer, and all 36 lymph nodes removed during the dissection did not show malignant cells. The three infiltrated lymph nodes in the right obturator fossa were confirmed to have extensive involvement by poorly differentiated cancer (**Figure 4**) with loss of MSH2 and MSH6 protein expression. There were no signs of endometriosis in the affected lymph nodes (e.g., neither hemosiderin nor signs of active bleeding).

Based on the finding of deficient mismatch repair proteins (dMMR), which is indicative of genetic disorders and Lynch syndrome in particular (31), genetic testing was recommended. This revealed an inherited deletion of the entire *EPCAM* gene up to exon 1-8 of the *MSH2* gene corresponding to Lynch syndrome. Patient underwent adjuvant combined chemotherapy consisting of paclitaxel and carboplatin (six cycles). As expected, after the resection of the right iliac veins, the patient developed transient lymphoedema of the right leg, which resolved spontaneously within a few weeks. No neurological sequelae were reported, as the integrity of the ventral part of the lumbosacral plexus was preserved. For the first 5 years, follow-up is planned to be in our gynecological oncology center with physical examination and pelvic and abdominal ultrasound imaging (controls every 3 months for the first 2 years; every 4 months in the third year; every 6 months for the fourth and fifth years). After the fifth year, yearly controls with a general gynecologist based on the patients individual surveillance plan. In addition, patient surveillance for lifetime risk of Lynch syndrome-associated colorectal cancer (30-73%) and gastric cancer (up to 18%) will be provided at a specialised centre for hereditary cancer syndromes, including annual colonoscopy and gastroduodenoscopy every 3 years (32).

Informed consent was obtained from the patient for publication of this article.

## 3 Discussion

This is the first case of nodal malignancy without evident gynecological or relevant medical history, documenting a young patient journey from the incidental finding of enlarged lymph nodes in the right obturator fossa to the diagnosis of a primary nodal, poorly differentiated endometrioid carcinoma after extensive diagnostic work-up and surgery. Patient is in complete clinical remission after 36 months postoperatively (**Supplementary Figure 5**).

In the diagnostic approach to lymphadenopathy, a vast array of diseases and drugs ought to be considered. The prevalence of nodal malignancy is approximately 0.4% in young population and 4% in adults (33). The region of the metastatic lymph nodes and its area of drainage can often be used to guide us toward a potential location of the pathology. Current evidence on evaluation and differential diagnosis of metastatic lymph nodes recommends a thorough medical history and physical examination as well as systematic work-up including biopsy of the most suspicious lymph node/-s, tumor marker profile, imaging, and other tests as appropriate (33). In this case, the young female patient had no relevant medical history besides multiple sclerosis treated with interferon-β. Her family history showed insignificant oncologic risk and her physical examination was unremarkable.

Ultrasound imaging offers high resolution of the pelvis with the endovaginal probe, permitting above else a meticulous scanning of the pelvic lymph node morphological and vascular architecture. This allows an accurate differentiation between benign and malignant transformations of lymph nodes with the possibility to perform ultrasound-guided biopsy (7, 10, 11). The Vulvar International Tumor Analysis (VITA) consensus opinion provides a guide for standardized assessment and description of lymph nodes using ultrasound (34). In this case, the expert sonographer identified three enlarged lymph nodes in the right obturator fossa with sonographic features of malignant changes such as subcapsular such as subcapsular tumor nodules contrasting against the residual non-infiltrated lymphoid tissue. The color Doppler demonstrated ring-shaped vessels around the subcapsular tumor nodules as well as transcapsular flow (**Figures 2**, **4** and **Videoclips 1**, **4**). In addition, two suspicious intraparenchymal liver lesions were described on the ultrasound (**Supplementary Figure S1** and **Videoclip 2**). An alternative technique to evaluate lymph node is PET-CT, which provides not only metabolic information but also a systemic staging. PET-CT confirmed the ultrasound findings showing high metabolism rate in the pelvic nodes but no enhanced metabolic activity in the hepatic lesions neither showed a possible primary tumor site.

To differentiate lymphoproliferative disease from secondary cancer metastases and to direct us to the possible primary source, tru-cut biopsy was performed, enabling a histologic diagnosis with high accuracy. In this case, poorly differentiated carcinoma was found in the lymph nodes with immunoprofile, suggesting endometrioid carcinoma differentiation. After an extensive histological examination of the specimen from hysterectomy and bilateral salpingo-oophorectomy, the primary source of tumor spread from uterus or adnexa was excluded and only nodal infiltration by endometrioid carcinoma was confirmed. Due to the result of histopathology and the young age, we have included MMR analysis to exclude Lynch syndrome. In tumor cells MLH1, PMS2 expression was retained in nuclei whereas MSH2 and MSH6 expression was lost, suggesting microsatellite instability (MSI, dMMR). These findings were highly suspicious of Lynch syndrome. Genetic testing was performed, which confirmed patient was a Lynch syndrome carrier of *EPCAM* gene deletion up to exon 1-8 of the *MSH2* gene. Lynch syndrome is inherited *via* a pathogenic germline variant in one of the four mismatch repair (MMR) genes *MLH1*, *MSH2*, *MSH6*, and *PMS2*. A second somatic hit affecting the remaining functional allele of the same MMR gene leads to DNA MMR deficiency. Thus, MMR deficiency is a major driving force in Lynch syndrome carcinogenesis.

Given the negative findings from hysterectomy and bilateral salpingo-oophorectomy, we considered primary malignant transformation of ectopic Müllerian tissue inside the lymph node. Regarding primary nodal malignant infiltration, to our knowledge, there are only a few case reports of Lynch syndrome–associated carcinoma with retroperitoneal lymph node infiltration (26, 28). Most similar case was reported by Koual et al. (28) on a 50-year-old patient with Lynch syndrome with previous history of a hyperplastic polyp who underwent prophylactic hysterectomy and bilateral salpingo-oophorectomy. During the surgery a nodule in the broad ligament was excised, revealing a nodal endometrioid adenocarcinoma within the pelvic lymph nodes, with no evident primary tumor (28).

Our case report represents the first case of nodal malignancy without evident gynecological pathology even after extensive work-up and with no possible link in the past history, unlike the Koual’s case where the endometrial hyperplasia may have caused a possible spillage or spread of undetected malignant cells in the lymph nodes.

Our case is also unique compared with the published literature in regard to the patient’s young age, absence of uterine abnormality, and the absence of a suspicious family history. We can hypothesize that the most likely origin was a primary infiltration from ectopic epithelial Müllerian tissue, which underwent malignant transformation. This could have happened by a possible spillage of endometrial tissue during the previous C-section or by the spread of occult endometriosis/endosalpingiosis reaching the hilum of the lymph nodes and/or the afferent lymphatic vessels. In this presented case, immunosuppression, due to inferferon-β therapy combined with genetic genotype, may have also amplified the cancerogenic cascade.

## 4 Conclusions

This is a rare case of a primary nodal poorly differentiated endometrioid carcinoma associated with Lynch syndrome. Ultrasound guided tru-cut biopsy of the suspected lymph node is essential for planning appropriate management. Immunohistochemical evaluation of MMR protein expression may help to detect Lynch syndrome-associated endometrioid carcinoma, allowing regular surveillance and immune checkpoint-based treatment options.

## Data availability statement

The original contributions presented in the study are included in the article/**Supplementary Material**. Further inquiries can be directed to the corresponding author.

## Ethics statement

Written informed consent was obtained from the individual(s) for the publication of any potentially identifiable images or data included in this article.

## Author contributions

DF: diagnosis and monitoring of the case, preparation of images and videos, writing and finalizing of the article. US: preparation of videos, graphics, writing and finalizing the article. NS: preparation of videos, graphics, writing and finalizing the article. TH: drafting the manuscript. AB: preparation of the article, especially the part focused on cross-section imaging, preparation of MRI images, PET-CT. RB: pathological evaluation of tru-cut biopsy, preparation of images. PD: pathological 2^nd^ expert review of all specimens, writing the article. KN: pathological evaluation of definitive specimen and writing the article. MV: oncological geneticist performing Lynch syndrome examination and writing the article. FF: gynecological oncologist performing the tru-cut biopsy under ultrasound control. RK: gynecological oncologist performing radical surgery. DC: gynecological oncologist performing radical surgery. TI-K: writing of the article, proofreading in English. All authors contributed to the article and approved the submitted version.

## Supplementary material

Supplementary Figure 1 Hepatic focal lesions. Ultrasound imaging demonstrates two inhomogeneous mostly hyperechoic lesions of 25 mm in the S4b segment of liver **(A)**; CECT shows hyperdense formation up to 25 mm in the S4b (arrow) without increased accumulation of 18F-FDG **(B)**; on MRI T2 weighted images with fat saturation, the lesions showed slightly hypointense deposits (arrow) **(C)**; and on the dynamic contrast sequence T1 weighted images with fat saturation, the lesions showed dynamic contrast enhancement in early and late phases (arrows) **(D)**. CECT (contrast enhanced computed tomography), 18F-FDG (18F-fluorodeoxyglucose), and MRI (magnetic resonance imaging). Ultrasound findings are also presented in **Videoclip 2**.

Supplementary Figure 2 Transvaginal tru-cut biopsy technique. In this case the needle penetration depth on the automated biopsy gun was chosen to be 15 mm due to the proximity of the large vessels **(A)**, the biopsy needle is inserted into a needle guide placed on the transvaginal probe; the guide fixes and determines the possible movement of the needle **(B)**; the stylet and cannula move automatically during the biopsy to avoid fragmentation of the sample; the end of the stylet penetrates the tissue, immediately behind it, there is a notch for biopsy sample collection, which is cut through the cannula **(C)**; the tip of the biopsy needle penetrating the target lesion is monitored on an ultrasound monitor during the biopsy **(D)**; and an 18G needle with a length of 30 cm was chosen for the transvaginal approach **(E)**. See also **Videoclip 3**.

Supplementary Figure 3 Morphological assessment of tru-cut biopsy sample. Non-fragmented core obtained from tru-cut biopsy (length 15 mm, width after fixation 1.2 mm) **(A)**, solid sheets of polygonal eosinophilic cells **(B)**.

Supplementary Figure 4 Immunoprofile of the tru-cut biopsy sample showed immunohistochemical positivity of cytokeratin 7 (CK7) **(A)** and of hepatocyte nuclear factor 1-beta (HNF1β) **(B)**.

Supplementary Figure 5 Timeline reporting the patient’s journey from diagnosis to final diagnosis, treatment, and follow-up.

Supplementary Video 1 Three bulky infiltrated lymph nodes can be identified in the right obturator fossa. In the first lymph node, a transcapsular flow and ring-shaped vessels can be seen with color Doppler imaging.

Supplementary Video 2 Ultrasound imaging showing hepatic focal lesions: Two inhomogeneous mostly hyperechoic lesions of 25 mm can be identified in the S4b segment of liver.

Supplementary Video 3 With real-time ultrasound monitoring, the needle tip can be seen to penetrate the target lesion and collect the biopsy as a hyperechogenic line. A total of three samples were taken.

Supplementary Video 4 Ultrasound imaging and histopathology showing residual non-infiltrated lymph node cortex (dashed yellow circles). The yellow filled circles on the ultrasound image and the arrows on the histopathology image show nodular tumor changes within the lymph node; in addition, the white dashed circle in the middle of specimen indicates necrotic changes within the tumor nodule.
