# Supplementary figures and images for: Primary retroperitoneal nodal endometrioid carcinoma associated with Lynch syndrome: A case report
Source: Front Oncol. 2023 Feb 21;13:1092044. doi: 10.3389/fonc.2023.1092044 (PMC9989303; doi:10.3389/fonc.2023.1092044)

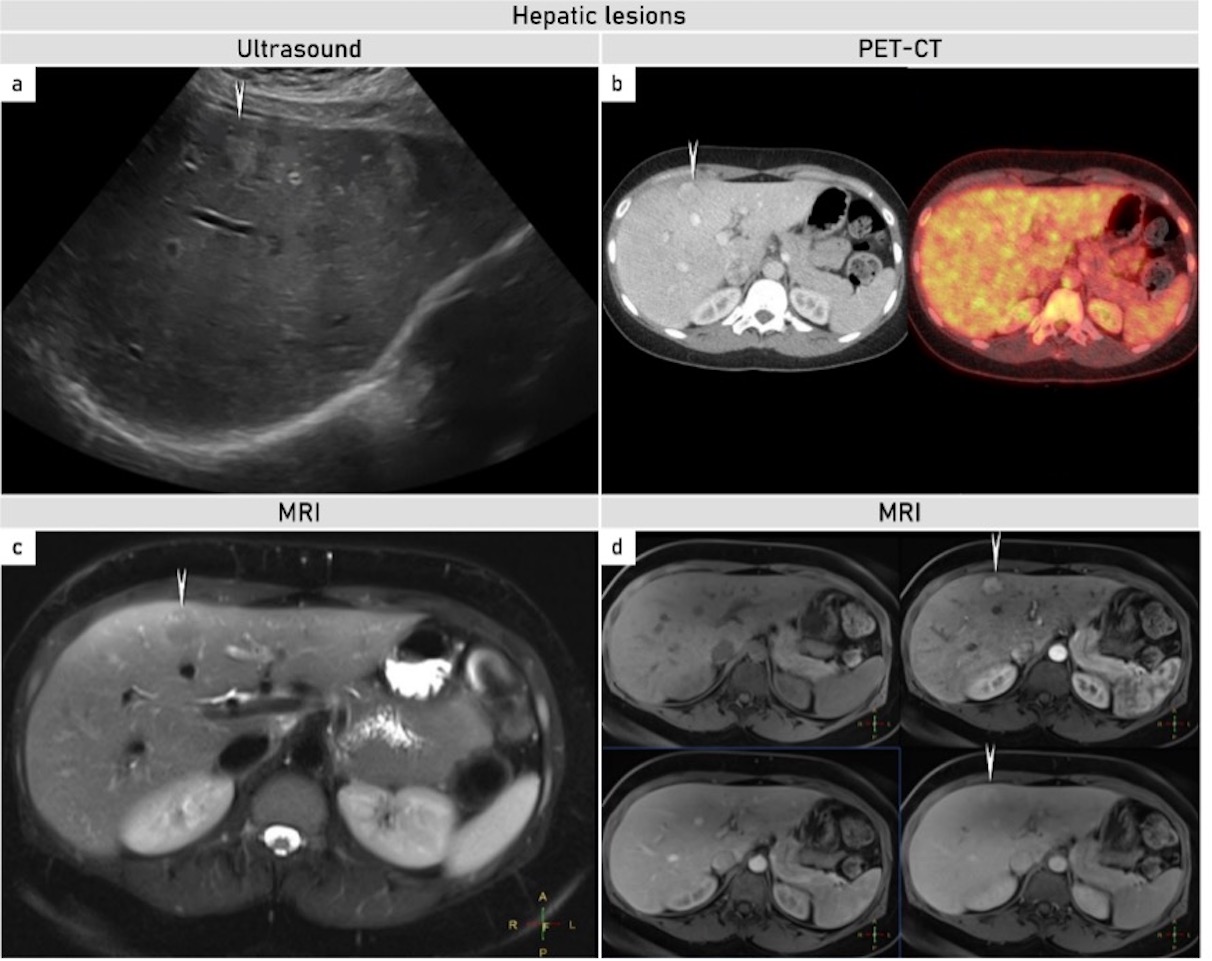

Supplement: Supplementary Figure 1 — Hepatic focal lesions. Ultrasound imaging demonstrates two inhomogeneous mostly hyperechoic lesions of 25 mm in the S4b segment of liver (A); CECT shows hyperdense formation up to 25 mm in the S4b (arrow) without increased accumulation of 18F-FDG (B); on MRI T2 weighted images with fat saturation, the lesions showed slightly hypointense deposits (arrow) (C); and on the dynamic contrast sequence T1 weighted images with fat saturation, the lesions showed dynamic contrast enhancement in early and late phases (arrows) (D). CECT (contrast enhanced computed tomography), 18F-FDG (18F-fluorodeoxyglucose), and MRI (magnetic resonance imaging). Ultrasound findings are also presented in Videoclip 2 . [file Image_1.jpeg]

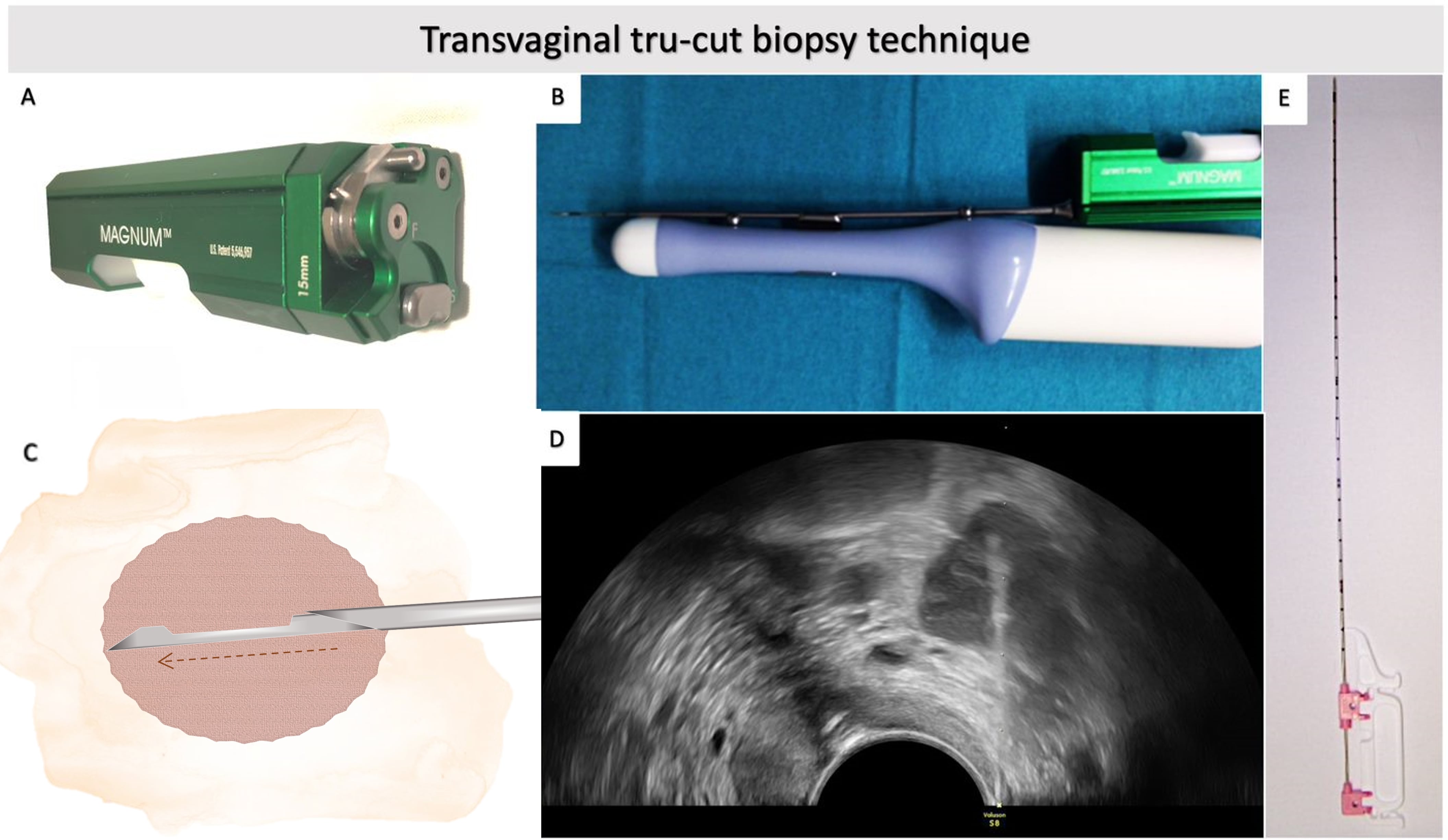

Supplement: Supplementary Figure 2 — Transvaginal tru-cut biopsy technique. In this case the needle penetration depth on the automated biopsy gun was chosen to be 15 mm due to the proximity of the large vessels (A), the biopsy needle is inserted into a needle guide placed on the transvaginal probe; the guide fixes and determines the possible movement of the needle (B); the stylet and cannula move automatically during the biopsy to avoid fragmentation of the sample; the end of the stylet penetrates the tissue, immediately behind it, there is a notch for biopsy sample collection, which is cut through the cannula (C); the tip of the biopsy needle penetrating the target lesion is monitored on an ultrasound monitor during the biopsy (D); and an 18G needle with a length of 30 cm was chosen for the transvaginal approach (E). See also Videoclip 3 . [file Image_2.jpeg]

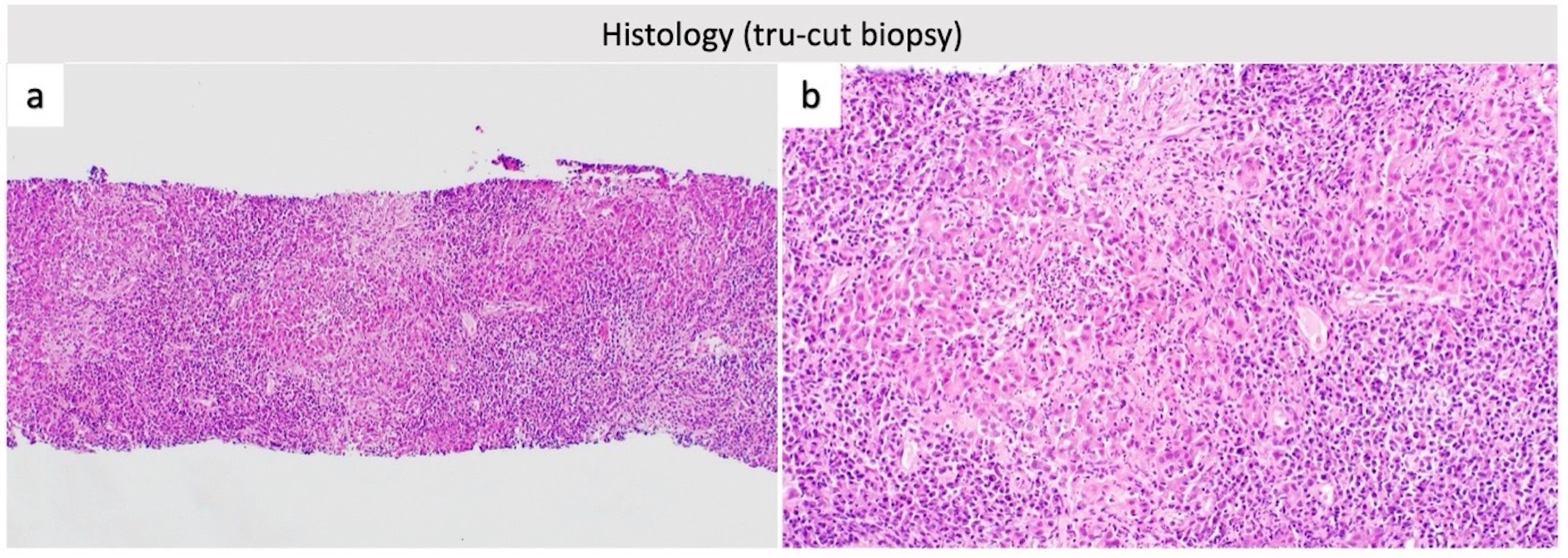

Supplement: Supplementary Figure 3 — Morphological assessment of tru-cut biopsy sample. Non-fragmented core obtained from tru-cut biopsy (length 15 mm, width after fixation 1.2 mm) (A), solid sheets of polygonal eosinophilic cells (B). [file Image_3.jpeg]

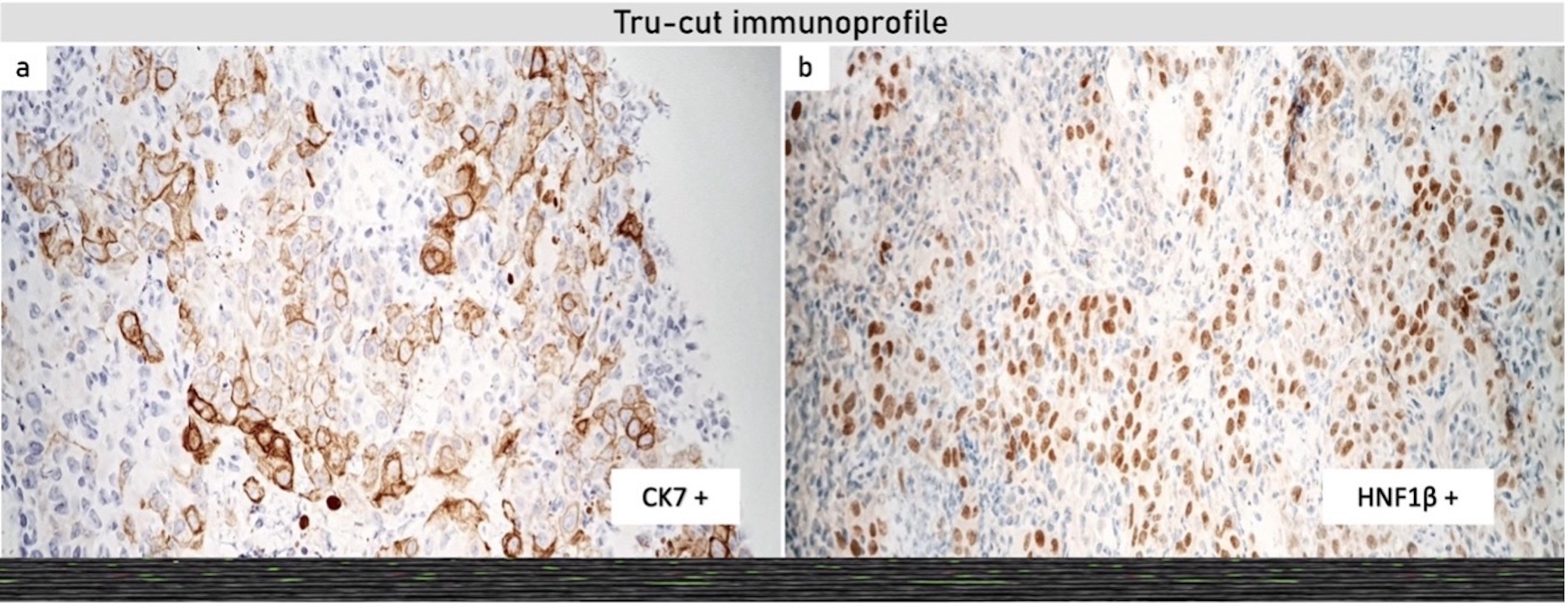

Supplement: Supplementary Figure 4 — Immunoprofile of the tru-cut biopsy sample showed immunohistochemical positivity of cytokeratin 7 (CK7) (A) and of hepatocyte nuclear factor 1-beta (HNF1β) (B). [file Image_4.jpeg]

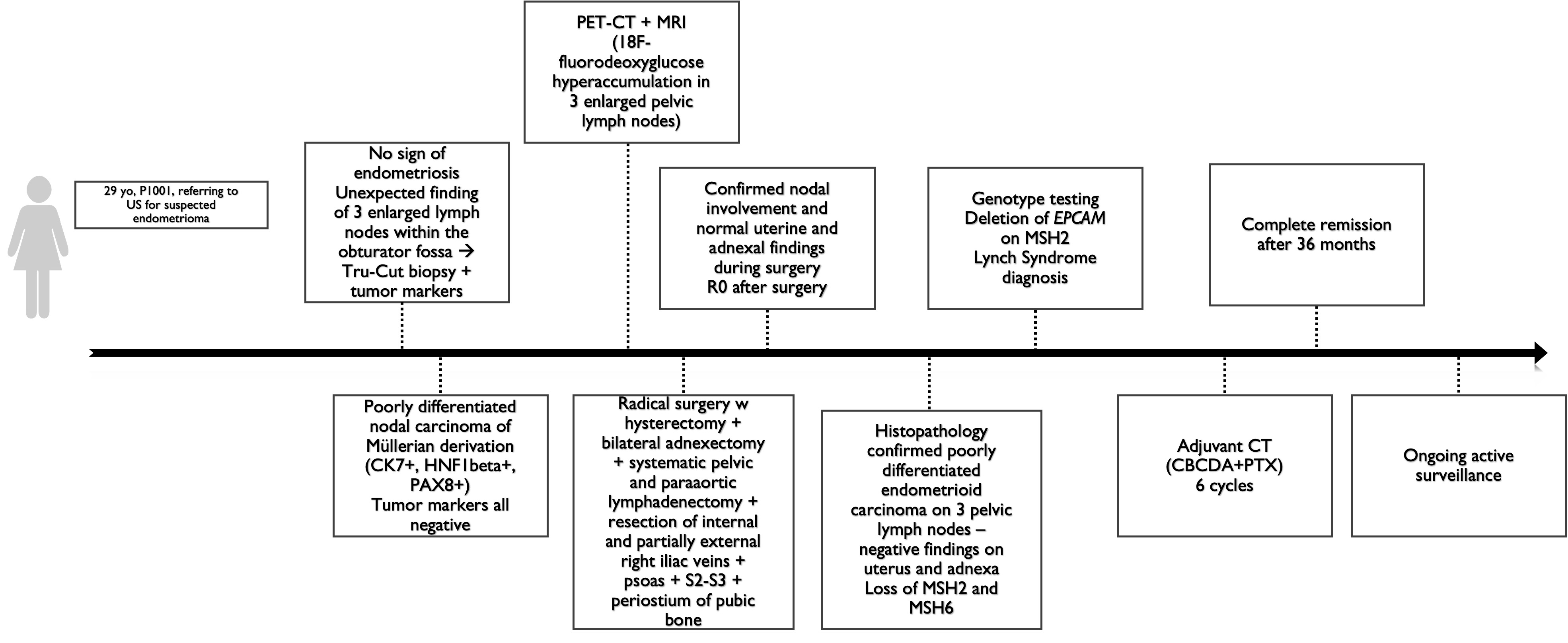

Supplement: Supplementary Figure 5 — Timeline reporting the patient’s journey from diagnosis to final diagnosis, treatment, and follow-up. [file Image_5.jpeg]
